# Prenatal allergen and diesel exhaust exposure and their effects on allergy in adult offspring mice

**DOI:** 10.1186/1710-1492-6-7

**Published:** 2010-05-11

**Authors:** Lin Corson, Huaijie Zhu, Chunli Quan, Gabriele Grunig, Manisha Ballaney, Ximei Jin, Frederica P Perera, Phillip H Factor, Lung-Chi Chen, Rachel L Miller

**Affiliations:** 1 Division of Pulmonary, Allergy and Critical Care Medicine, Department of Medicine, Columbia University College of Physicians and Surgeons, New York, New York 10032, USA; 2 Environmental Health Sciences, New York University, Tuxedo, New York 10987, USA; 3 Columbia Center for Children's Environmental Health, Mailman School of Public Health Columbia University, New York, New York 10032, USA

## Abstract

**Background:**

Multiple studies have suggested that prenatal exposure to either allergens or air pollution may increase the risk for the development of allergic immune responses in young offspring. However, the effects of prenatal environmental exposures on adult offspring have not been well-studied. We hypothesized that combined prenatal exposure to Aspergillus fumigatus (*A. fumigatus*) allergen and diesel exhaust particles will be associated with altered IgE production, airway inflammation, airway hyperreactivity (AHR), and airway remodeling of adult offspring.

**Methods:**

Following sensitization via the airway route to *A. fumigatus *and mating, pregnant BALB/c mice were exposed to additional *A. fumigatus *and/or diesel exhaust particles. At age 9-10 weeks, their offspring were sensitized and challenged with *A. fumigatus*.

**Results:**

We found that adult offspring from mice that were exposed to *A. fumigatus *or diesel exhaust particles during pregnancy experienced decreases in IgE production. Adult offspring of mice that were exposed to both *A. fumigatus *and diesel exhaust particles during pregnancy experienced decreases in airway eosinophilia.

**Conclusion:**

These results suggest that, in this model, allergen and/or diesel administration during pregnancy may be associated with protection from developing systemic and airway allergic immune responses in the adult offspring.

## Background

Epidemiological studies and murine models suggest that prenatal environmental exposures can enhance the risk for developing asthma in the offspring [1,2]. In humans, prenatal exposures to air pollutants such as environmental tobacco smoke (ETS) and polycyclic aromatic hydrocarbons (PAHs) have been shown to be associated with asthma-related outcomes in young children [1,3,4]. In mice, prenatal exposure to residual oil fly ash was associated with increased airway hyperresponsiveness, allergic inflammation, and elevated immunoglobulin (Ig) E and IgG_1 _in the ovalbumin (OVA) sensitized offspring by age 16-37 days [2]. Offspring mice of mothers that were exposed to diesel exhaust particles (DEP) and immunologically inert substances such as titanium dioxide and carbon black particles during pregnancy also were more susceptible to developing airway hyperreactivity and inflammation following ovalbumin sensitization, suggesting that the mechanism to induce enhanced risk for asthma by inert substance exposure is not antigen-specific [5]. Most recently, a diet high in methyl donors during pregnancy was associated with a greater degree of airway allergic inflammation that was transmitted to a third generation of mice. These changes were associated with altered DNA methylation of Runt-related transcription factor 3 (RUNX3), implicating epigenetic regulation in the transmission of an asthma-related phenotype across generations[6].

Alternately, some prenatal exposures have induced protection from the asthma phenotype. Lipopolysaccharide (LPS or endotoxin) administered prenatally to mice led to the development of lower anti-OVA IgE and IgG_1 _levels, eosinophilia in BAL fluid, and reduced phorbol 12-myristate 13-acetate (PMA), inomycin, and OVA-induced T helper (Th) 2 cytokine production in the offspring [7,8]. In epidemiological studies, prenatal exposure to farms, sources of endotoxin exposure, was associated as well with childhood protection from asthma, hay fever, and atopic sensitization [9]. Furthermore, mice whose mothers were immunized with *Dermatophagoides pteronyssinus *(*D. pteronyssinus*) allergen prior to mating developed significant decreases in total and anti-*D. pteronyssinus *IgE, IgG_1_, IgG_2a _and IgG_2b _levels upon resensitization in comparison to offspring of unexposed mice [10]. Hence, in some models, prenatal allergen exposure may confer immunological tolerance or protection from atopy in the offspring.

Despite these advances, many key questions still need to be elucidated. These include questions about the effects of airborne prenatal exposures to toxins of concern in the urban environment, as well as their possible long-term adverse effects on adult offspring. Our objective was to determine the effects of concomitant and chronic aerosolized prenatal exposure to allergen and diesel exhaust particles, two environmental exposures implicated in inner city asthma [11,12], on phenotypes that develop in adult offspring mice. Our strategy was to employ the *A. fumigatus *mouse model that induces strong allergic responses via the airway route in the absence of adjuvants and, hence, arguably better mimics clinical asthma [13]. Diesel exhaust was routed through an exposure chamber and administered during pregnancy [14,15]. We hypothesized that combined prenatal exposure to *A. fumigatus *and diesel exhaust particles would be associated with altered IgE, airway inflammation, airway hyperreactivity (AHR), and airway remodeling in adult mice offspring.

## Methods

### *A. fumigatus *sensitization

**Figure 1:**
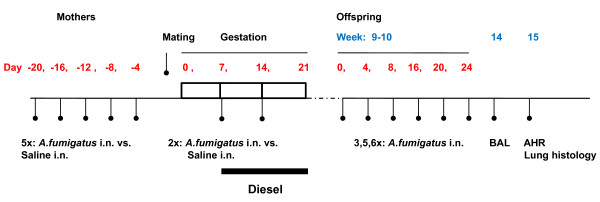
**Experimental protocol**. Adult females received 5 dosages of *A. fumigatus *or saline, 20, 16, 12, 8, and 4 days prior to mating. During the second and third weeks of pregnancy, mothers received diesel exhaust particle exposure Monday through Friday plus *A. fumigatus *or saline on days 7 and 14. *AHR: Airway hyperreactivity BAL: Bronchoalveolar lavage i.n: intranasal 3×: 3 doses of A. fumigatus 5×: 5 doses of A. fumigatus 6×: 6 doses of A. fumigatus*

Six week old wild-type female and male BALB/c mice were obtained from Jackson Laboratories (Bar Harbor, ME). Males and females were housed separately prior to mating. All animals were housed at New York University (NYU) animal facility (Tuxedo, NY) and fed a commercial pellet mouse feed. Mice were lightly anesthetized with isoflurane (2% inhaled). Intranasal application of *A. fumigatus *(62.5 ug) (Hollister-Stier Co., Spokane, WA; measured endotoxin dose < 0.16 EU/ml: Endotoxin Testing Service, Cambrex Bio Science Walkersville, Inc, MD) in 50 ul of saline or saline vehicle alone was administered five times, four days apart, beginning 20 days prior to mating. Pregnant mice were treated again with *A. fumigatus *or saline on day 7 and 14 after mating. Offspring were separated from their mothers at 21 days of age. At 9-10 weeks of age, all offspring were treated with either five or six dosages of *A. fumigatus *each dose four days apart (Figure 1). All experimental procedures were approved by IACUCs at Columbia University and New York University.

### Diesel exposure

Diesel exhaust was produced by a 5500-watt single cylinder diesel engine generator (Yanmar YDG 5500EE-6EI; Osaka, Japan) that contained a 418-cc displacement engine (Model LE100EE-DEGY6), as described [15,16]. The engine was operated at a maximum engine load condition using Number 2 on-road ultra-low-sulfur diesel fuel delivered from a local gas station (SOS Fuels, Tuxedo Park, NY) and 15W/40 engine oil (SAE, 15W/40, Delo400, Chevron Products Company, San Ramon, CA). The diesel exhaust particles (DEP) were diluted to a desirable level through a serial dilution system with HEPA-filtered ambient air, and routed to a 1 m^3 ^flow-through exposure chamber where mice were exposed. Pregnant mice were exposed for 5 hours (average 5.18 hours) a day, Mondays through Fridays, to DEP or HEPA (high efficient particle) filtered ambient air (as negative control) in parallel during the second and third weeks of pregnancy (Figure 1).

The mass concentrations of the DEP in the exposure chamber were recorded every 20 minutes using a real-time Personal DataRam (PDR) aerosol monitor (Model: PDR1000, MIE Inc., Bedford, MA). DEP also were collected daily onto Teflon filters (Gelman Teflo, 37 mm, 0.2 *u*m pore; Gelman Sciences, Ann Arbor, MI) for subsequent gravimetric analyses. Particle size distributions were measured with a Wide-Range Particle Spectrometer (0.01 to 10 μm, WPS, MSP Corp., Shoreview, MN). The average particle concentration was 1.09 mg/m^3^. The DEP atmosphere had a count median aerodynamic diameter of 80 nm, and a mass median aerodynamic diameter of 152 nm.

### Blood collection and measurement of IgE, IgG_1_, IgG_2a_

Sera were obtained from adult female mice immediately prior to the first dose of *A. fumigatus*, and 2 days following the fifth dose of *A. fumigatus *versus saline prior to mating. Sera were obtained from their offspring prior to the first, and one day after the third, fifth, and sixth (last) dose of *A. fumigatus*. Sera were aliquoted and frozen. Total Ig levels were measured by ELISA using isotype specific capture antibodies for IgE, IgG_1 _and IgG_2a _(BD PharMingen, Franklin Lakes, NJ), following a previously described protocol [17]. Briefly, 96 well microtiter plates were coated with rat anti-mouse IgE, IgG_1 _or IgG_2a_. Sera were diluted 1:20 for IgE, 1:10,000 for IgG_1_, and 1:100 for IgG_2a_. Biotin labeled rat-mouse IgE, IgG_1 _and IgG_2a _along with AKP (alkaline-phosphatase) Streptavidan (BD Pharmingen, Franklin Lakes, NJ) were used for detection. Specimens were run in duplicate and averaged.

### BAL and cellular analysis

Mice were euthanized at median age 12.5 weeks and bronchoalveolar lavage was performed three times on each mouse with 1 ml of phosphate buffered saline (PBS) 24 hours after the last allergen challenge. Lavage fluid was centrifuged at 4°C 1500 rpm for 5 minutes. Cell pellets were resuspended in 1 ml of phosphate buffered saline. Slides were prepared using a cytocentrifuge (Cytospin;Shandon) at 500 rpm for 5 minutes then stained with Wright-Giemsa stain (Sigma-Aldrich, St. Louis, MO). 100 cells total were counted for each sample from 10 randomly chosen viewing fields and total eosinophil, lymphocyte, macrophage and neutrophil counts were quantified by a blinded reader.

### Airway hyperreactivity, lung histology and assessment for remodeling

At median age 15.5 weeks, additional mice were anesthetized and intubated with a 20 g catheter inserted directly into the trachea via a neck dissection, then placed on a flexivent ventilator. A nebulizer attached to the flexivent apparatus exposed mice to increasing concentrations of methacholine at 8, 16, 32, 64 mg/ml (Sigma-Aldrich, St. Louis, MO). Airway resistance was determined by the flexivent-apparatus (SCIREQ, Montreal, Quebec, Canada) [18]. The shape of each dose-response curve was examined to determine whether each mouse responded to aerosolized methacholine, as described elsewhere [19]. Data obtained from aberrant curves were discarded prior to data analysis.

Immediately subsequent to the AHR testing, lungs were inflated and stored in 10% formalin. Lungs were paraffin-embedded and sections were stained with hematoxylin and eosin (Sigma-Aldrich, St. Louis, MO). Under blinded conditions, each lung was scored for perivascular inflammation, peribronchial inflammation and arterial remodeling as previously described [13]. For perivascular and peribronchial inflammation, lungs were scored semiquantitatively as follows: 1 = normal with very few inflammatory cells bordering the arteries or airways; 2 = scattered inflammatory cells surrounding the artery or airway up to two rings in depth; 3 = cell cuffs or clusters of inflammatory cells surrounding the artery or airway three rings or more in depth. Arterial remodeling was scored as follows: 1 = normal; 2 = thickened vascular wall with intact lumen and circular media; 3 = obstructed lumen and thickened wall lined with disorganized layers of cells.

### *A. fumigatus*-specific T cell proliferation

Splenocytes (1 × 10^6^/ml) were seeded in triplicate in 96 well plates and treated with *A. fumigatus *(Hollister-Stier, Spokane, WA) at 0, 20 ug/ml or 40 ug/ml and CD3 10 μg/ml (BD, Franklin Lakes, NJ) and incubated with 5% CO_2 _for five days at 37°C. ^3^H-thymidine uptake was assessed on day 5 as described [20].

### Statistical analysis

One-way analysis of variance (ANOVA) was used to compare mean differences across treatment groups followed by Tukey HSD test except where noted. Nonparametric rank order correlations were used to compare continuous data (eg. IgE levels and eosinophil counts) between treatment groups. Differences were considered statistically significant at p < 0.05.

## Results

### Effects of *A. fumigatus*, diesel exhaust exposure, on adult female pregnant mice

**Figure 2:**
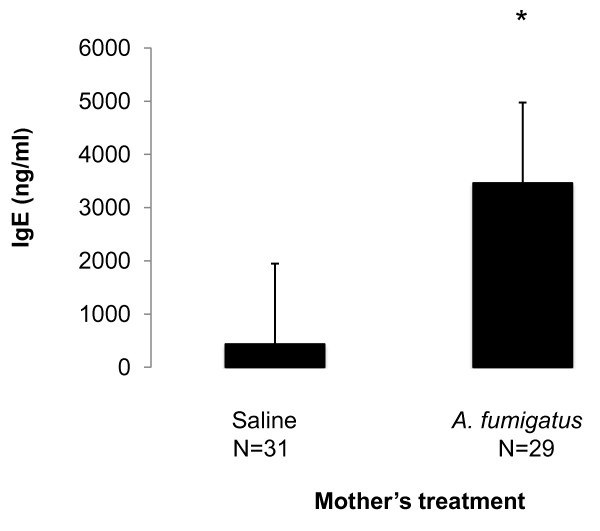
**IgE levels following sensitization of mothers to *A. fumigatus***. IgE levels were measured in adult females after 5 doses of *A. fumigatus *and immediately prior to mating. *p < 0.0001, two tailed Mann-Whitney test

Adult female mice sensitized to *A. fumigatus *developed higher total IgE levels than those treated with only vehicle saline solution (p < 0.0001, MannWhitney U, Figure 2). Higher eosinophil absolute counts and percentage of total white blood cells also were detected among sensitized adult female mice immediately prior to euthanasia on bronchoalveolar lavage (p < 0.0001 for both). There were no significant differences in airway inflammation across treatment groups following breeding. The airway inflammation score in retired female breeder mice treated with saline only (mean score: 1.7) was greater than the expected baseline of 1.0-1.4 observed in 2-3 month old experimental wild type mice [13,21]. To ascertain whether prenatal exposure to *A. fumigatus *and/or DEP also can induce airway remodeling in the mothers, as reported in wildtype C57BL/6 mice, pulmonary arterial remodeling was assessed across treatment groups [13]. Significant differences in arterial remodeling across groups were not detected.

### Ig induction in offspring after three, five and six doses of *A. fumigatus*

**Figure 3:**
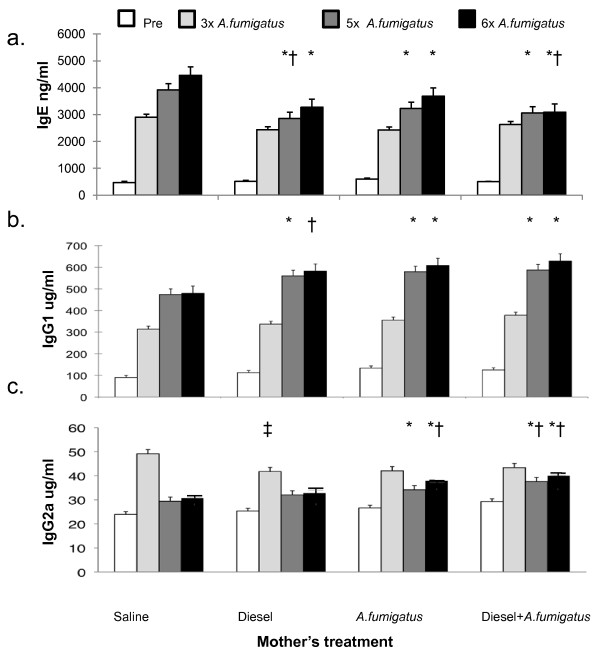
**Ig induction in offspring after three, five and six doses of *A. fumigatus***. a) IgE was reduced after the fifth and sixth (p < 0.0001 on ANOVA), but not third (p = NS, ANOVA), doses among offspring mice whose mothers were exposed to either *A. fumigatus *or diesel exhaust particles or both. *p < 0.01, when compared to saline alone by Tukey HSD. † p < 0.05, when compared to *A. fumigatus *alone by Tukey HSD b) IgG_1 _was greater after the fifth, sixth (p < 0.0001 on ANOVA), but not third (p = NS, ANOVA), doses among mice whose mothers were exposed to either *A. fumigatus *or diesel exhaust particles or both. *p < 0.01, when compared to saline alone by Tukey HSD. †p < 0.05, when compared to saline alone by Tukey HSD. c) IgG2a was greater after the fifth, sixth (p < 0.0001 on ANOVA) dose among mice whose mothers were exposed to *A. fumigatus *or diesel exhaust particles plus *A. fumigatus*. *p < 0.01, when compared to saline alone by Tukey HSD. Levels also were greater among offspring of mothers that were exposed to both diesel exhaust particles and *A. fumigatus *when compared to offspring of mothers treated either with diesel exhaust or *A. fumigatus *alone, p < 0.01. †p < 0.01, when compared to diesel exhaust particles alone by Tukey HSD. ‡ p < 0.05 on ANOVA and when compared to saline alone by Tukey HSD. Sample sizes corresponding to the figures vary as follows: Saline 11-14; Diesel 11-15; *A. fumigatus*: 8-18; Diesel and *A. fumigatus *13-25.

Adult offspring from mothers who received *A. fumigatus *or DEP alone, or *A. fumigatus *and DEP together, developed lower levels of total IgE when assessed after the fifth dose of *A. fumigatus *compared to offspring from mothers treated with saline only prior to mating (p < 0.0001 ANOVA, Figure 3a). In addition, IgE levels from offspring from mice exposed to DEP alone were lower than those from offspring from mice exposed to *A. fumigatus *alone (p < 0.05 Tukey HSD test). Adult offspring from mothers who received *A. fumigatus *or DEP alone, or DEP and *A. fumigatus *together, developed lower IgE levels compared with levels from offspring whose mothers received saline alone when assessed after the sixth dose of allergen treatment as well (p < 0.0001 ANOVA). In addition, IgE levels from adult offspring of mice that were treated with DEP and *A. fumigatus *were lower than those from offspring of mice that were treated with *A. fumigatus *alone (p < 0.05, Tukey HSD test). Significant differences in IgE levels were not apparent after the third dose of *A. fumigatus*

In contrast, offspring from mothers exposed to *A. fumigatus*, DEP, or both DEP and *A. fumigatus*, developed greater IgG_1 _levels compared to offspring of mothers treated with saline. This effect was significant after the fifth and sixth, but not third, doses of *A. fumigatus *treatment (p < 0.001 ANOVA, Figure 3b).

Further, offspring from mothers exposed to *A. fumigatus*, or both DEP and *A. fumigatus*, developed greater IgG_2a _levels compared to offspring of mothers treated with saline alone when assessed after the fifth dose of *A. fumigatus *(p < 0.001 ANOVA). Also, offspring from mothers exposed to both DEP and *A. fumigatus*, developed greater IgG_2a _levels compared to offspring of mothers exposed to either DEP or *A. fumigatus *alone (p < 0.01 Tukey HSD test). Adult offspring from mothers who received *A. fumigatus *alone, or DEP and *A. fumigatus *together, developed greater IgG_2a _levels compared with levels from offspring whose mothers received saline or DEP alone after the sixth dose of allergen as well (p < 0.0001 ANOVA). In contrast, after the third dose of *A. fumigatus*, a reduction in IgG_2a _was detected among offspring from mice exposed to DEP compared with those treated with saline (p < 0.05, Tukey HSD, Figure 3c).

### Prenatal exposure to *A. fumigatus *and diesel exhaust particles was associated with reduced airway eosinophilia in adult offspring

**Figure 4:**
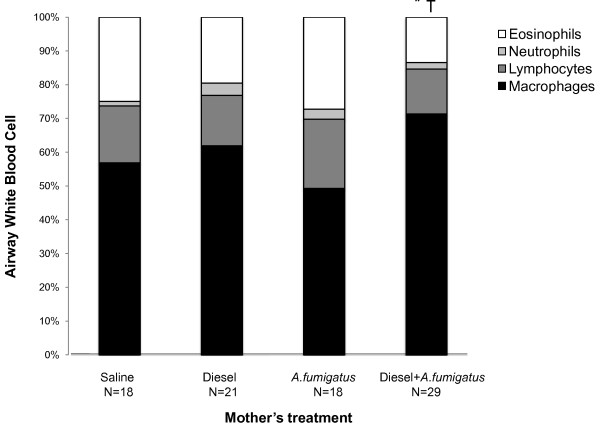
**Differential airway cell counts in offspring after five and six doses of *A. fumigatus***. Eosinophil counts were significantly decreased (and macrophages significantly increased) among offspring from mothers following diesel exhaust and *A. fumigatus *compared to offspring of mothers treated with saline alone, * p < 0.0002 on ANOVA and p < 0.05 by Tukey HSD or with *A. fumigatus *alone, † p < 0.0003 on ANOVA and p < 0.01 by Tukey HSD.

Adult offspring from mothers that received both *A. fumigatus *and DEP developed significantly less airway eosinophilia (mean eosinophil count 13.24 ± 2.04%) compared to offspring from mothers that had received *A. fumigatus *(26.44 ± 2.89%, p = 0.01, Tukey HSD) or saline (23.83 ± 3.33%, p = 0.05, Tukey HSD) alone. The first result (*A. fumigatus *and DEP lower than *A. fumigatus*) was replicated when examining absolute numbers of eosinophils (p < 0.001 on ANOVA and p < 0.01 by Tukey HSD). Adult offspring from mothers that received both *A. fumigatus *and DEP also developed higher levels of macrophage counts compared to offspring of mothers that had received *A. fumigatus *(p = 0.01, Tukey HSD) or saline (p = 0.05, Tukey HSD) alone (Figure 4). Airway eosinophil counts did not correlate with IgE levels measured at any of the time points (Spearman rank correlation R-value = -0.055 after the third dose, 0.019 after the fifth dose and -0.082 after the sixth dose, p = nonsignificant (NS) for each). Airway eosinophil counts also did not correlate with IgG_1 _levels after the fifth (R-value = 0.216, p = NS) or sixth dose (R-value = -0.185, p = NS).

### Effects on perivascular, peribronchial airway inflammation, airway remodeling, and airway hyperreactivity

**Figure 5:**
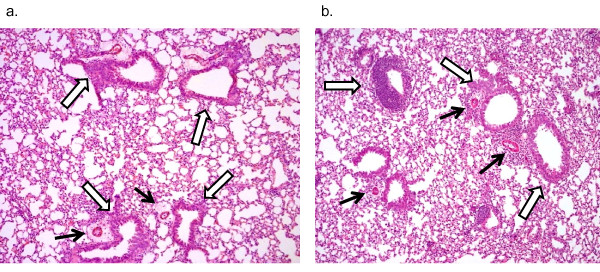
**Histological changes in offspring from mothers exposed to saline or *A. fumigatus***. a.) Representative histology from lungs of offspring whose mother was treated with saline following 5 doses of *A. fumigatus *starting at 9-10 weeks. The photomicrograph was taken from a lung section stained with Hematoxylin and Eosin. White arrows point to perivascular and peribronchial inflammation, black arrowheads point to mild arterial muscularization. b.) Representative histology from lung of offspring whose mother was treated with *A. fumigatus *following 5 doses of *A. fumigatus *starting at 9-10 weeks. The photomicrograph was taken from a lung section stained with Hematoxylin and Eosin. White arrows point to perivascular and peribronchial inflammation, black arrowheads point to arterial muscularization. No differences were observed among offspring of mice that received diesel exhaust, particles when compared to offspring of mice that received saline. No differences were observed among offspring of mice that received diesel and *A. fumigatus*, compared to *A. fumigatus *alone.

To ascertain whether systemic changes in Ig levels and airway changes in eosinophil counts were associated with additional histological and physiological alterations, perivascular, peribronchial airway inflammation, airway remodeling, and airway hyperreactivity were assessed in the adult offspring following prenatal exposure to diesel exhaust and/or *A. fumigatus*. On histological examination, examples of perivascular and peribronchial inflammation and arterial muscularization were detected among adult offspring of mice exposed to *A. fumigatus *when compared to offspring of mice exposed to saline (Figure 5). Differences were not observed among offspring from mice that were exposed to DEP, when compared to offspring of mice that received saline, or among offspring of mice that received DEP and *A. fumigatus*, compared to *A. fumigatus *alone.

**Figure 6:**
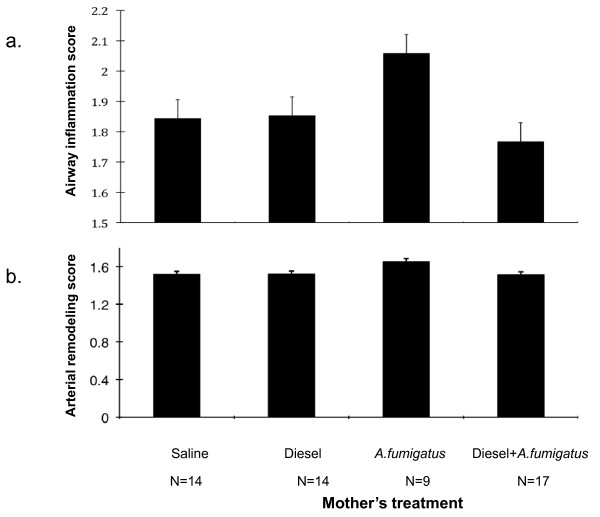
**Perivascular, peribronchial airway inflammation and airway remodeling in offspring**. a. Perivascular, peribronchial airway inflammation in offspring of *A. fumigatus*. Composite scores were obtained following 5 or 6 doses of *A. fumigatus*. Significant differences across groups were not detected. p = 0.11 on ANOVA. Perivascular and peribronchial inflammation were scored as follows [13]: 1 = normal with very few inflammatory cells bordering the arteries or airways; 2 = scattered inflammatory cells surrounding the artery or airway up to two rings in depth; 3 = cell cuffs or clusters of inflammatory cells surrounding the artery or airway three rings or more in depth. b. Arterial remodeling in offspring. Offspring from mothers treated during pregnancy as outlined in Fig. 1 were exposed to *A. fumigatus *intranasally with 5-6 doses starting at 9-10 weeks of age. Arterial remodeling was scored as described [13] and in Figure 3b. Significant differences across groups were not detected. p = 0.183 on ANOVA. Arterial remodeling was scored as follows [13]: 1 = normal; 2 = thickened vascular wall with intact lumen and circular media; 3 = obstructed lumen and thickened wall lined with disorganized layers of cells.

However, using an established semi-quantitative scoring system [13], only a nonsignificant trend in airway inflammation was observed among mice that were treated with *A. fumigatus *when compared to mice whose mothers were treated with saline alone (1.84 ± 3.74 saline vs. 2.05 ± 0.07 *A. fumigatus *mean airway inflammation score ± SE, p = 0.11 ANOVA)(Figure 6a). Also, we were unable to detect a correlation between total airway inflammation scores and IgE levels measured at any of the 3 time points (R-value = 0.096 after the third dose, 0.016 after the fifth dose and -0.077 after the sixth dose, p = NS for each) (Figure 6b). Correlations between inflammation score and IgG_1 _(R-value = 0.228 after the fifth dose, 0.222 after the sixth dose, p = NS for each) were not detected either.

To ascertain whether prenatal exposure to *A. fumigatus *and/or DEP can induce airway remodeling in adult sensitized offspring, pulmonary arterial remodeling was assessed in the mice offspring. Offspring from mothers who received *A. fumigatus *and/or DEP during pregnancy did not exhibit significant differences in the degree of arterial airway remodeling compared to offspring of mothers who received saline (Figure 6b). Arterial remodeling scores between mothers and their offspring exhibited a borderline correlation (spearman R = 0.269, p = 0.096).

In addition, differences in AHR across any treatment groups were absent (data not shown).

### Prenatal exposure to *A. fumigatus*, diesel was not associated with altered *A. fumigatus*-induced T cell proliferation

To determine whether prenatal exposure to *A. fumigatus *and/or DEP would be associated with altered antigen-specific T cell proliferation in the offspring, splenocytes were tested following induction with several doses of *A. fumigatus *or anti-CD3. We found that antigen-specific proliferation in the adult offspring was not affected by *A. fumigatus *or DEP exposure administration to the mother (data not shown).

## Discussion

These results suggest that exposures to *A. fumigatus *prior to and during pregnancy were associated with diminished IgE production and airway eosinophilia. The latter occurred following prenatal exposure to both *A. fumigatus *and diesel. The parallel increases in IgG levels suggest that the antibody responses were specific to IgE. These findings indicate that prenatal exposure to *A. fumigatus*, may be associated with protection from systemic and airway allergic immune responses in adult offspring.

While these results may appear contradictory to several studies that show prenatal sensitization (i.e. to ovalbumin) is associated with greater allergic immune responses in the offspring [5], they are consistent with a few studies that suggest prenatal environmental exposures can suppress the subsequent risk for an asthma-related phenotype or induce tolerance. For example, transfer of antigens from mother to mouse pup via breast milk has induced oral tolerance and antigen-specific protection from allergic airway disease [22]. In addition, prenatal sensitization to *D. pteroynissinus *was associated with lower total and *D. pteroynissinus*-IgE levels in the offspring. Similar to our model, exposure to allergen prior to mating reduced allergen sensitization in the offspring at the humoral level [10]. In more recent work by the same group, the induction of allergic sensitization versus tolerance following prenatal exposure to ovalbumin was determined to be dependent on the dose and timing of exposure. Specifically, prenatal oral exposure to high dose ovalbumin was associated with lower ovalbumin-IgE in the pups at age 3 days following immunization. The effect was transient, and subsequent increases in ovalbumin-IgE levels were detected at age 25 days. Also, the effect was best observed if the ovalbumin treatments occurred during the first week of pregnancy. However, pups born to mothers who received prenatal oral administration of low dose ovalbumin showed similar decreases in ovalbumin IgE levels and antigen-specific T cell proliferation, but this tolerogenic effect was more sustained[23]. Prenatal LPS exposure also was associated with suppression of IgG_1 _and IgE and reduction of interleukin (IL)-5 and IL-13 in splenic mononuclear cells [7]. Besides endotoxin, prenatal oral exposure to the chemical bisphenol A has been associated with preferential T helper (Th) 1 immune responses in sensitized adult offspring mice [24]. Hence, in the model described here, prenatal exposure to *A. fumigatus *and diesel may have timed or dosed so as to favor establishment of tolerance instead of allergic sensitization.

While postnatal exposure to mold has been associated with greater asthma severity or emergency room visits for asthma [24-27], recent studies suggest that exposure to mold allergen after birth may be protective. These include two cross sectional cohort studies that found higher levels of fungal β(1,3)-glucans, fungal extracellular polysaccharides and endotoxin in dust collected from mattresses used by asymptomatic children age 5-13 compared with those used by atopic children who wheezed [28,29]. It has been hypothesized that fungal products besides the associated allergens, such as dust endotoxin, extracellular polysaccharides (EPS) and glucans may induce immunologic protection from the development of atopic disease [28,29]. As another example, inner-city children, aged 2 to 6 years old, living in homes with either comparatively low ( < 2 μg/g Mus m 1) or high (> 29.9 μg/g Mus m 1) dust levels of mouse allergen developed attenuated humoral responses in comparison to those who lived in homes with a medium level of measured allergen in their dust (2-7.9 μg/g Mus m 1). This work also suggests that the development of protection from allergic sensitization occurs and may be related to the dose of allergen exposure. However, the extent of allergic sensitization, rather than the measured level of allergen detected in dust or delivered via aerosol, tends to be more strongly associated with allergy symptoms in an inner-city cohort study [30].

EPA has estimated occupational DEP exposures to range from 39 - 191 *μ*g/m^3 ^for railroad workers, 4 - 748 *μ*g/m^3 ^for firefighters, and 7 - 98 *μ*g/m^3 ^for public transit workers and airport crews [31]. So while the chronic administration of inhaled diesel exhaust particles may have mimicked some natural physiological conditions in this model, the levels employed are higher than most urban environments and some occupational ones. Diesel exposure has been associated with upregulation of the allergic immune responses and airway remodeling in both animal and human studies [32,33]. However, the independent and synergistic effects of prenatal diesel exposure administered in this manner and reported here seem small. Adult offspring from mothers who received DEP alone, or *A. fumigatus *and DEP together, developed lower levels of total IgE, and greater levels of IgG_1_, when assessed after the fifth and sixth dose of allergen. Paradoxically, after the third dose of *A. fumigatus*, a reduction in IgG_2a _was detected among offspring from mice exposed to DEP compared with those treated with saline. Also, adult offspring of mothers that received both *A. fumigatus *and DEP developed significantly less airway eosinophilia compared to offspring of mothers that had received *A. fumigatus *alone. Combined, these results suggest that prenatal DEP exposure independently may have conferred some protection against allergic immune responses in the adult offspring in this model. These findings were unexpected, especially given previous research using the engine byproduct residual oil fly ash as the air pollutant that induced greater airway eosinophilia and hyperreactivity in the OVA-sensitized offspring [2]. It is unclear whether the disparate phenotypes are related to the antigens administered (ovalbumin vs. *A. fumigatus*), components and dose of the air pollutants, strain of mouse, or age of the offspring following allergen sensitization (less than 5 weeks vs. 9-10 weeks).

Associations between prenatal exposure to DEP and/or *A. fumigatus *and airway arterial remodeling in adult offspring were not statistically significant, with only mild changes detected during histological examination. Exposure to *A. fumigatus *has been shown to exacerbate an asthma phenotype in rats by aggravating Th2 inflammation, increasing AHR, and inducing airway remodeling [34]. Previously, a few mouse models have induced airway remodeling following repeated and chronic OVA exposure and the recruitment of eosinophils, IL-13 and profibrotic cytokines have been implicated [35-37]. Our group previously showed that adult C57BL/6 mice treated intermittently with *A. fumigatus *for a prolonged period of time developed remodeling of small to medium sized pulmonary arteries [13]. In another mouse model, maternal exposure to cigarette smoke during pregnancy was found to be associated with airway remodeling in the offspring at ten weeks of age, as demonstrated by increases in airway smooth muscle thickness, collagen deposition and house dust mite induced increases in neutrophils, mast cells and goblet cell hyperplasia [38]. From a cohort study, offspring of mothers who smoked during pregnancy developed permanent vascular damage that was not apparent in offspring of non-smoking mothers [39]. This current study, to our knowledge, represents the first examination of the effects of these environmental exposures on airway remodeling across generations of mice.

Several plausible mechanisms may explain how prenatal exposures may help modulate the development of allergic and/or airway immune response in the offspring. It has been reported that antigen-specific T cell and B cell immune responses in the fetus can occur distinctly from those of the mother, as demonstrated by our group in response to vaccination against influenza [40]. In addition, previous reports also suggested that the transfer of allergy across the placenta may be regulated by the transfer of cytokines that may influence the development of allergic sensitization. Supportive data include a murine model that demonstrates that administration of anti-IL-4 can inhibit allergic immune responses from sensitization to OVA in the offspring [41]. In addition, combined inhaled diesel exhaust and *A. fumigatus *exposure has been shown to induce hypermethylation of multiple CpG sites of the interferon-gamma (IFNg) promoter and hypomethylation of one CpG site of the IL-4 promoter with associated changes in IgE levels, suggestive of the contribution of epigenetic regulation following environmental exposures [16]. These mechanisms seem plausible in light of recent associations between prenatal exposure to polycyclic aromatic hydrocarbons or high methyl diet and DNA methylation of asthma candidate genes [6,42]. However, these reports do not directly explain mechanistically how prenatal exposure to *A. fumigatus*, or diesel, may induce *protection *from allergy in the offspring, especially in light of past data that suggest *A. fumigatus *induces greater, not repressed, Th2 cytokine production [13]. In one study, offspring of Balb/c mice whose mothers were tolerized with ovalbumin by means of oral application of antigen also were protected from the development of an asthma-like phenotype as late as 8 month after birth. This protection was blocked by inhibition of IFNγ [42]. Transfer of IgG antibodies from suckling or from the placenta has also been shown to suppress IgE following prenatal exposures to egg albumin [22]. Rats whose mothers were immunized with egg albumin during pregnancy experienced a diminished capacity to develop IgE and enhanced IgG responses during early adulthood, and these results were replicated when separate offspring were administered small quantities of immune serum 3 weeks after birth[43].

Some limitations of this study merit discussion. First, we used only one strain of mice to obtain the above findings even though it has been shown that when comparing acute injury responses, such as airway remodeling, patterns are unique to different strains[44]. Also, the use of a mouse model does not give us a comprehensive representation of what occurs after prenatal sensitization in humans because we are not able to accurately replicate some human behaviors such as smoking and diet. Relatively higher inflammation scores among retired mothers and their adult offspring are difficult to explain, but could be a result of accumulated lung injury due to dust from dirty bedding in breeder cages or stress (personal observation).

## Conclusion

In conclusion, our results indicate that *A. fumigatus *administration during pregnancy resulted in protection from systemic and airway allergic responses. Prenatal diesel exhaust particle exposure also was associated with reduced IgE levels and suppressed airway eosinophilia in the adult offspring. These results suggest that prenatal environmental exposures can induce exert systemic and airway immune changes in the adult offspring related to respiratory disease. These results highlight the need to consider the health effects of prenatal exposures on offspring, even through adulthood.

## List of abbreviations

AHR: airway hyperreactivity; AKP: alkaline-phosphatase; BAL: bronchoalveolar lavage; BHR: bronchial hyperresponsiveness; DEP: diesel exhaust particles; EPS: extracellular polysaccharides; ETS: environmental tobacco smoke; HDM: house dust mite; IFN: interferon; Ig: immunoglobulin; IL: interleukin; in: intranasal; LPS: lipopolysaccharide; NS: non-significant; NYU: New York University; OVA: ovalbumin; PAH: polycyclic aromatic hydrocarbon; PMA: phorbol 12-myristate 13-acetate; RUNX3: Runt-related transcription factor 3; Th: T-helper

## Competing interests

The authors declare that they have no competing interests.

## Authors' contributions

LL carried out the experimental work, performed some of the statistical work, and drafted the manuscript. HZ carried out the experimental work. CQ carried out all exposure related experiments. GG participated in the design of the study and advised on the experimental work. MB carried out a significant proportion of the experimental work. XJ worked with CQ administer the diesel exposure. FPP participated in the design of the study and advised on the experimental work. PHF participated in the design of the study and advised on the experimental work. LCC participated in the design of the study and advised on the experimental work. RLM conceived of the study, performed the statistical work, and participated in its design and coordination and drafted the manuscript. All authors read and approved the final manuscript.
